# Intraoperative cone-beam computed tomography to secure the surgical margin in pulmonary wedge resection for indistinct intrapulmonary lesions

**DOI:** 10.1016/j.xjtc.2022.01.028

**Published:** 2022-02-23

**Authors:** Kazuhiro Ueda, Masaya Aoki, Go Kamimura, Nobuhiro Imamura, Takuya Tokunaga, Soichi Suzuki, Masami Sato

**Affiliations:** Department of General Thoracic Surgery, Graduate School of Medical and Dental Sciences, Kagoshima University, Sakuragaoka, Kagoshima, Japan

**Keywords:** lung cancer, subsolid tumor, sublobar resection, cone-beam computed tomography, hybrid operating room, CBCT, cone-beam computed tomography, CT, computed tomography

## Abstract

**Objective:**

The objective of this study was to use cone-beam computed tomography (CBCT) for intraoperative imaging of a pulmonary wedge resection line that contributes to securing the required surgical margin in patients undergoing thoracoscopic surgery for indistinct intrapulmonary lesions.

**Methods:**

Data of 16 consecutive patients with potentially impalpable intrapulmonary lesions were retrospectively reviewed. Preoperatively, we simulated a rhomboidal cut line on the surface of a 3-dimensional lung model with reference to multiplanar reconstruction computed tomography images. Intraoperatively, we imaged the rhomboid on the real lung surface using trial and error adjustment with CBCT. Wedge resection was performed thoracoscopically by stapling along the outline of the rhomboid.

**Results:**

The mean consolidation diameter and mean distance between the tumor and the visceral pleura were 2 mm and 11 mm, respectively. In all cases, we only performed single CBCT scanning to localize the rhomboid on the real lung surface. The mean radiological distance between the approximate location and the correct location was 8 mm (range, 0-34 mm). Wedge resection was successful with a mean surgical margin of 11 mm (range, 7-16 mm), without conversion to anatomical resection or open conversion. This simulation was also helpful for planning port placement for the use of an autostapler.

**Conclusions:**

We established a novel procedure for imaging the cut line on the lung surface with intraoperative CBCT, which facilitated the performance of wedge resection with the required surgical margin in patients with potentially impalpable intrapulmonary small lesions. Our method might be beneficial for patients and surgeons because it can be applied without preoperative intervention.

**Figure:**
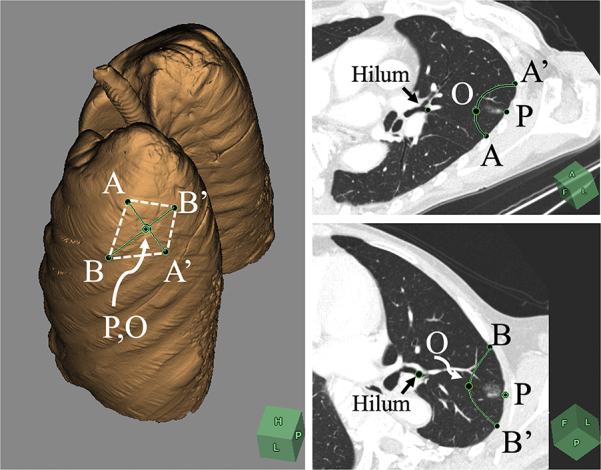
Imaging of a cut line on the lung for securing the surgical margin length in a hybrid OR.

**Central Message**

Cone-beam computed tomography-guided imaging of a cut line on the lung facilitated successful wedge resection with a required surgical margin in patients with indistinct subpleural lesions.

**Perspective**

We introduced a novel procedure for radiological image-based simulation of pulmonary wedge resection, which helped secure a safe margin length in patients with indistinct subpleural lesions. This procedure is beneficial for surgeons, in planning stapling of the lung in a case-specific manner, and for patients, because it does not involve preoperative intervention.

Thoracoscopic pulmonary wedge resection for intrapulmonary small lesions, particularly those with a ground glass component, is challenging because such lesions are likely difficult to locate using thoracoscopic vision or finger palpation. Various methodologies have been reported for the preoperative localization of indistinct intrapulmonary lesions, including the transthoracic insertion of hook wires,^1^ microcoils,^2^ or blue dye.^3^ However, these modalities can cause complications, such as pulmonary hematoma, hemoptysis, and, more seriously, systemic air embolism.^1^^,^^4^^,^^5^ In contrast, bronchoscopic marking with blue dye^6^ or indocyanine green^7^ is relatively safe, but is troublesome because it requires the combined use of bronchoscopy and computed tomography (CT) or fluoroscopy. In addition, the instilled dye can spread widely, compromising the accuracy of the localization of pulmonary small lesions. Preoperative intervention also leads to patient stress. To solve these issues, many investigators reported the usefulness of cone-beam CT (CBCT) in intraoperative localization of the indistinct small intrapulmonary nodules.^8^, ^9^, ^10^ Nonetheless, although these methods are certainly helpful for identifying the tumor location, they do not help to secure the surgical margin.

We have developed a CT-based preoperative simulation of pulmonary wedge resection. In this model, we can draw a cut line on a 3-dimensional volume-rendering lung model. The cut line can be imaged accurately on the real lung with the intraoperative use of CBCT images. We hypothesized that intraoperative imaging of the cut line on the lung with CBCT would contribute to the resection of indistinct intrapulmonary tumors with an appropriate surgical margin.

## Methods

### Patients

This was a retrospective review of 16 patients who underwent pulmonary wedge resection for potentially impalpable intrapulmonary lesions with CBCT guidance between May 2020 and May 2021. This study was approved by our institutional review board (number 170088; approval date, August 7, 2017). Written informed consent was obtained from all patients. The characteristics of the 16 patients (male, n = 7; female, n = 9; mean age, 68.4 years) are shown in Table 1. Basically, CBCT-based marking for a small indeterminate pulmonary nodule was considered when the distance to the nearest pleural surface was more than 5 mm in cases of lung nodules of 10 mm or less in diameter.^11^ We also used CBCT in patients with predominant ground glass lesions if the lesions were separated from the pleura. The final diagnosis was primary lung adenocarcinoma in 11 patients, metastatic lung cancer in 4, and benign lesion in 1. All 11 patients with primary lung cancer showed ground glass attenuation on preoperative CT images. The lesion was located in the right upper lobe in 2 patients, right middle lobe in 2, right lower lobe in 7, left upper lobe in 3, and the left lower lobe in 2. The mean whole tumor diameter was 7.8 mm, the mean solid diameter was 2.4 mm, and the mean distance between the visceral pleura and tumor was 11.3 mm.

**Table 1:** Patient characteristics and radiopathological findings of the lung tumor

Case	Age, years	Sex	Tumor location	Final diagnosis	Maximum diameter, mm	Solid diameter, mm	GGO	Depth, mm
1	81	M	LS1+2	AIS	11	0	Yes	5
2	67	F	RS6	MIA	12	0	Yes	5
3	52	M	LS9	Meta	7	7	No	15
4	67	F	RS9	MIA	11	0	Yes	3
5	83	M	RS10	Benign	10	10	No	7
6	67	M	LS3	Meta	5	5	No	21
7	76	F	RS6	AAH	5	0	Yes	7
8	68	F	RS8	AIS	6	0	Yes	17
9	64	F	RS4	AIS	7	0	Yes	3
10	66	F	RS3	MIA	4	4	No	20
11	71	F	LS1+2	MIA	9	0	Yes	23
12	73	F	RS10	AIS	10	0	Yes	15
13	71	M	RS6	Meta	4	4	No	11
14	65	M	RS10	MIA	11	4	Yes	4
15	56	M	LS6	AIS	8	0	Yes	10
16	68	F	RS5	Meta	4	4	No	15

*GGO*, Ground glass opacity; *M*, male; *LS*, left segment; *AIS*, adenocarcinoma in situ; *F*, female; *RS*, right segment; *MIA*, minimally invasive adenocarcinoma; *Meta*, metastatic lung cancer; *AAH*, atypical adenomatous hyperplasia.

### Theoretical Basis of Wedge Resection

**Figure 1:**
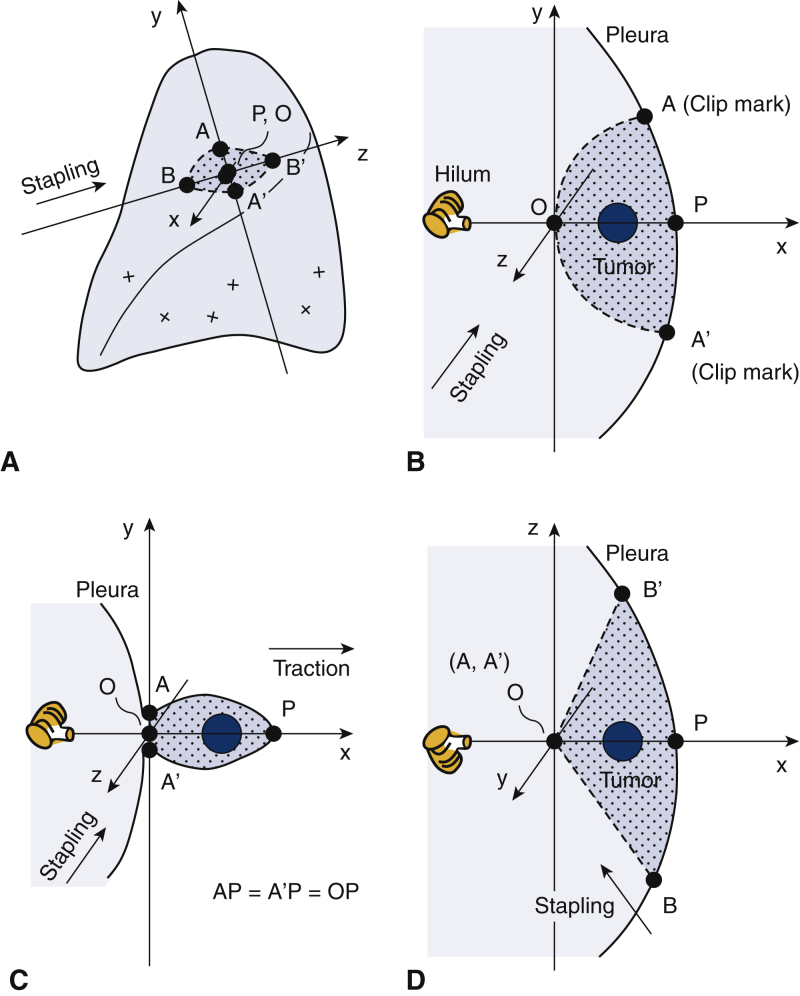
Schematic illustration of a cut line (*dashed line*) for an intrapulmonary tumor located at left anterior segment (A). The cut line (ABA'B') can be approximated to a rhomboid. The direction of the cut line (BB') is the stapler pathway. The x-axis passes through the center of the tumor and the orifice of the anterior segmental bronchus (B and C). The y-axis passes through the deepest point (*O*) of the wedge resection (B and C). The z-axis also passes through point O and is parallel to the direction of the stapler pathway (D). Point P is defined as the intersection of the x axis and the pleura (B). In the x-y plane, point A and A' is placed on the pleura (OP = AP = A'P) (B). During wedge resection, point P is pulled in the x-axis direction, and point A, O, and A' are cut simultaneously by the stapler (C and D). In the x-z plane, point B and B' are placed on the pleura so that the stapler passage (BOB') is adequate with regard to the surgical margin.

Figure 1, *A* shows a schematic illustration of the cut line for an intrapulmonary tumor located at the left anterior segment. This cut line (dashed line) can be approximated to a rhomboid. The direction of the cut line is dependent on the site at which the stapler is inserted. The x-axis passes through the center of the tumor and the orifice of the anterior segmental bronchus (Figure 1, *B* and *C*). The y-axis passes through the deepest point (O) of the wedge resection (Figure 1, *B* and *C*). The z-axis also passes through point O and is parallel to the direction of the stapling device that is to be inserted (Figure 1, *D*). Point P is defined as the intersection of the x-axis and the pleura (Figure 1, *B*). In the x-y plane, point A and A' are placed on the pleura (OP = AP = A'P; Figure 1, *B*). During wedge resection, point P is pulled in the x-axis direction, and point A, O, and A' are cut simultaneously by the stapler (Figure 1, *C* and *D*). In the x-z plane, point B and B' are placed on the pleura so that the stapler passage is adequate with regard to the surgical margin.

### Preoperative Simulation of Wedge Resection

**Figure 2:**
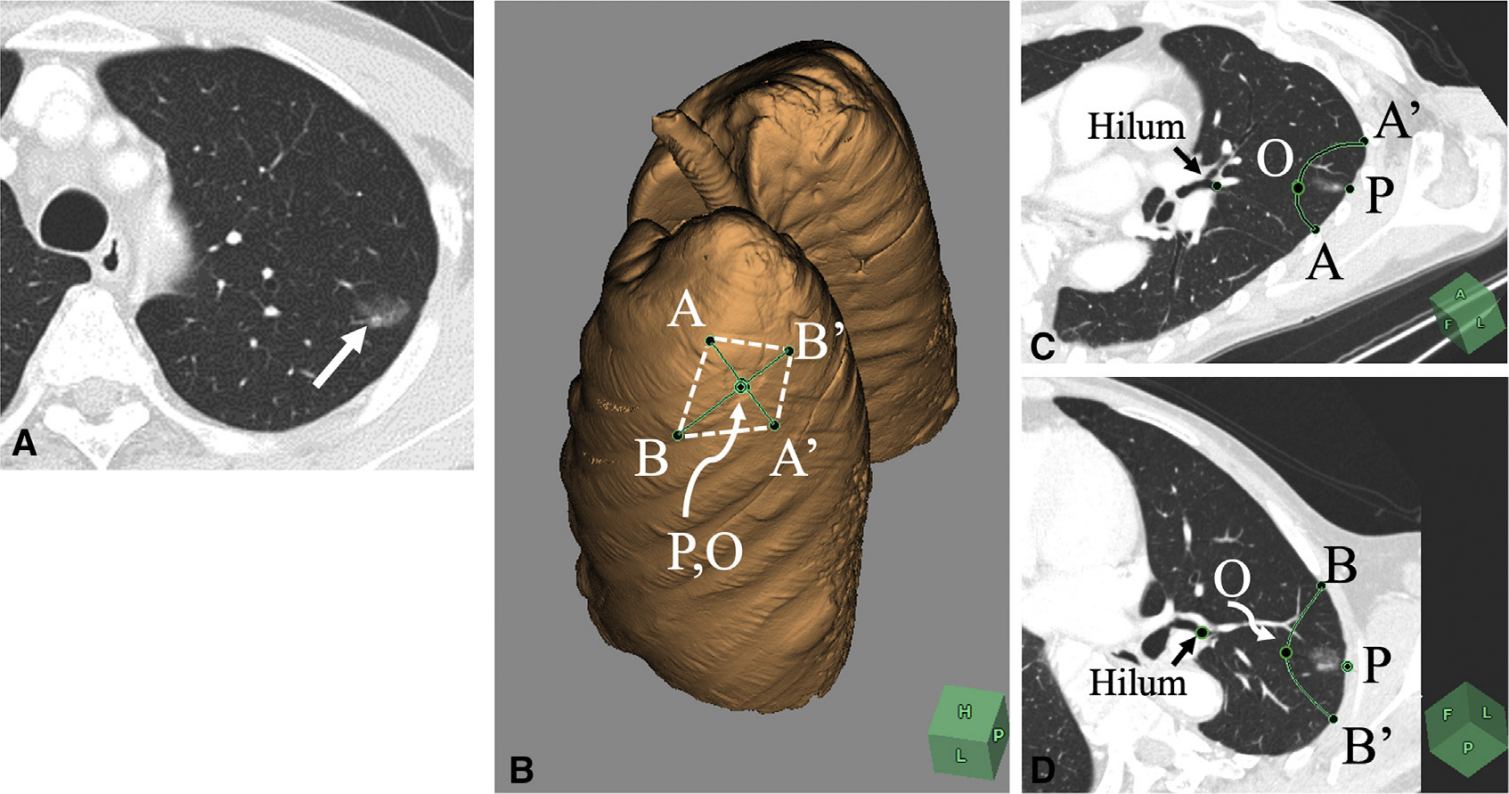
A representative case of preoperative simulation of wedge resection of the left upper lobe for subsolid nodule in the apicoposterior segment (A). By placing points A, A', B, and B' on the pleura on the basis of the theoretical basis of wedge resection, a rhomboidal cut line can be drawn on the 3-dimensional volume rendering lung model (B). The x-y plane (C) and the x-z plane (D) were generated using multiplanar reconstruction. *O*, deepest point of the wedge resection.

Figure 2 shows a representative case of preoperative simulation of wedge resection of the left upper lobe for a subsolid nodule in the apicoposterior segment (Figure 2, *A*). By placing points A, A', B, and B' on the pleura on the basis of the theoretical basis of wedge resection (described previously), a rhomboidal cut line can be drawn on the 3-dimensional volume-rendering lung model (Figure 2, *B*). The x-y plane (Figure 2, *C*) and x-z plane (Figure 2, *D*) were generated using multiplanar reconstruction. These images were analyzed using a commercially available simulation software program (Synapse Vincent; FujiFilm).

### Intraoperative CBCT

After general anesthesia with endobronchial intubation using a double-lumen tube, scans were acquired using a C-arm CBCT (Artis Zeego; Siemens Healthcare GmbH) during end-inspiratory breath-holding with an airway pressure of 15 cm H_2_O. Patients were fixed at lateral decubitus position on the dedicated surgical bed in the hybrid operating room. Scans were obtained with a 6-second acquisition protocol (6-s DynaCT body; Artis Zeego; Siemens Healthcare GmbH). Scanned data were also analyzed using Synapse Vincent.

### Intraoperative Imaging of the Cut Line

**Figure 3:**
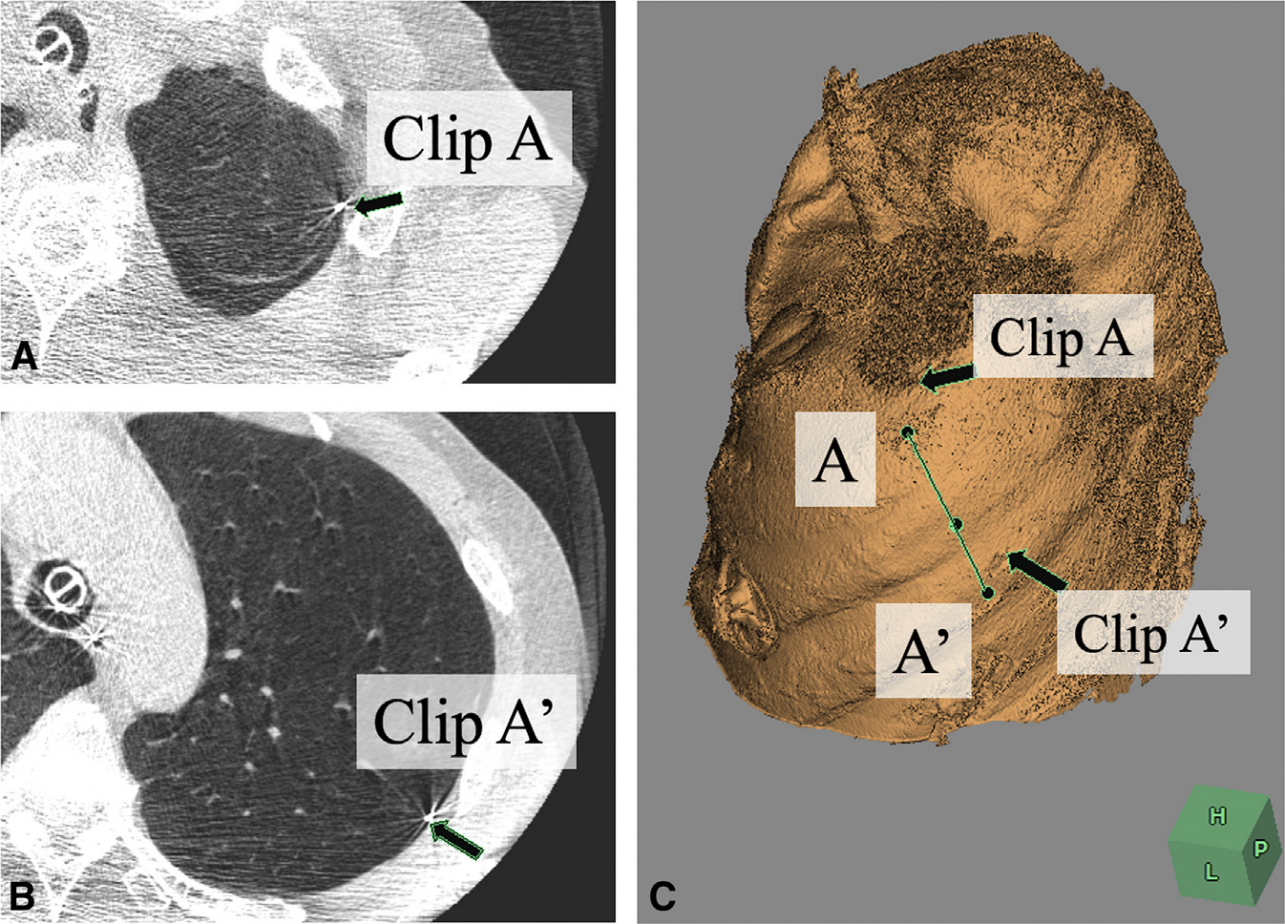
Intraoperative cone-beam computed tomography obtained after clipping of approximated points A and A' under bilateral ventilation (A and B). The correct points A and A' were determined on the basis of the multiplanar reconstruction images on cone-beam computed tomography, and the discrepancies between the clipped point A and the correct point A, as well as the clipped point A' and the correct point A' were measured (C).

**Figure 4:**
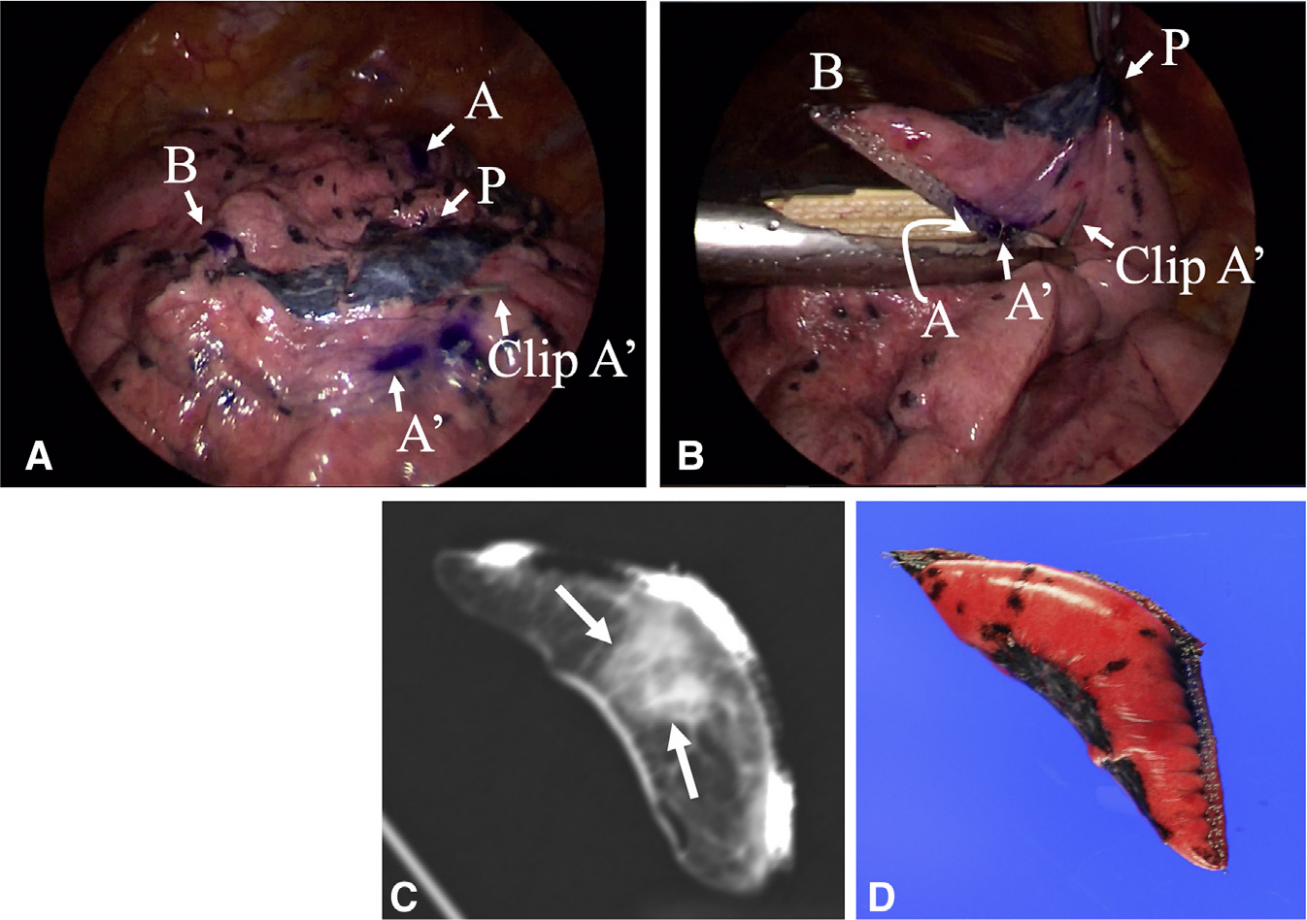
After assessment with cone-beam computed tomography, the correct points A and A' are eventually marked on the pleura using an ink pen (A). the correct sites of points A and A', as well as points B and B' can be marked on the deflated lung, using an ink pen and a ruler (A), which enables imaging of the cut line. During surgery, point P is pulled in the opposite direction to the hilum (apicoposterior bronchus), and the left upper lobe is cut from point B to point B' via point A and A' using a stapler (B). If the tumor is impalpable after careful palpation of the resected specimen, the specimen is scanned using conventional computed tomography after inflating the specimen with air, to confirm if the tumor is indeed resected (*arrows* indicate lung tumor), as well as to confirm if the surgical margin length is adequate (C). Note that the intrapulmonary lesion is located at the center of the resected specimen (D).

Theoretically, the rhomboidal cut line can be imaged on the real lung if points A and A' are accurately located on the lung surface because points B and B' can be determined on the basis of the location of points A and A'. Thus, we approximated points A and A', with reference to the distances from some anatomical landmarks, including the pulmonary fissure and apex of the upper lobe, and the approximated points A and A' were marked with surgical vascular clips. Subsequently, CBCT was taken using bilateral ventilation. The correct locations of points A and A' were determined on the basis of the multiplanar reconstruction images on CBCT (Figure 3), and discrepancies between clipped point A and correct point A, as well as clipped point A' and correct point A' were measured. Correct points A and A' were eventually marked on the pleura with an ink pen (Figure 4, *A*). The differences in the scale between the inflated lung and the deflated lung can be determined by comparing the distance between the 2 clips on CBCT images with that on the real deflated lung. On the basis of the scale adjustment, the correct locations of points A and A', as well as points B and B' can be marked on the deflated lung, using an ink pen and a ruler (Figure 4, *A*), which enables imaging of the cut line. During surgery, point P is pulled in the opposite direction to the hilum (apicoposterior bronchus), and the left upper lobe is cut from point B to point B' via point A and A' using a stapler (Figure 4, *B*). If the tumor was impalpable after careful palpation of the resected specimen, the specimen is scanned using conventional CT after inflating the specimen with air, to confirm that the tumor was resected, as well as to confirm that the length of the surgical margin was adequate (Figure 4, *C*).

### Definition of Required Surgical Margin

According to a previous study, a surgical margin length that is equal to or greater than the maximum tumor diameter is recommended for patients with non–small cell lung cancer of <2 cm in maximum diameter, to avoid positive stump cytology.^12^ On the basis of the recommendation, we defined the required surgical margin as equal to the maximum tumor diameter, measured using preoperative CT. Therefore, point O, representing the deepest point of wedge resection, was placed where the distance from the tumor was equal to the maximum tumor diameter.

### Validation of Surgical Margin Length

Postoperatively, the resected specimens were soaked in 10% formalin neutral buffer solution for 48 hours. After removing the stapler needles from the staple line, the specimen was cut into pieces for macroscopic evaluation of the margin length (after shrinkage). We previously compared the surgical margin length of the resected specimen inflated with air on intraoperative CT with the margin length after shrinkage, measured on a formalin-fixed specimen.^13^ As a result, the radiologically measured margin length was 20% longer than the margin length after shrinkage. On the basis of the result, we estimated the margin length before shrinkage (estimated margin length) by multiplying the margin length after shrinkage by 1.2. Eventually, we determined whether or not the estimated margin length satisfied the required surgical margin length (maximum tumor diameter), which was measured on inflated lung.

### Surgical Stump Cytology

We routinely performed irrigation of any cartridges used in pulmonary stapling to make a rapid cytological diagnosis of surgical stump positivity.^14^ We generally make additional resection if the irrigation cytology was positive.

### Statistical Analysis

The numerical data are expressed in mean and range. We did not use statistical comparisons because this study was comprised of a single arm.

## Results

Video 1 provides a summary of thoracoscopic wedge resection of the left upper lobe using CBCT guidance. Intraoperatively, there were no adverse events related to the CBCT-based localization of the cut line or the subsequent wedge resection. In addition, there were no postoperative complications during the 30-day observation period. The mean length of postoperative chest tube drainage was 1.4 days (range, 1-4 days), and the mean length of postoperative hospital stay was 7.7 days (range, 3-14 days). Because this study was on the basis of our initial experience, CBCT was performed for test scanning just before the operation and a confirmatory scan was optionally performed after resection, in addition to the single acquisition for the purpose of imaging the cut line (the total acquisition was 2 times in 4 patients and 3 times in 12 patients).

**Figure 5:**
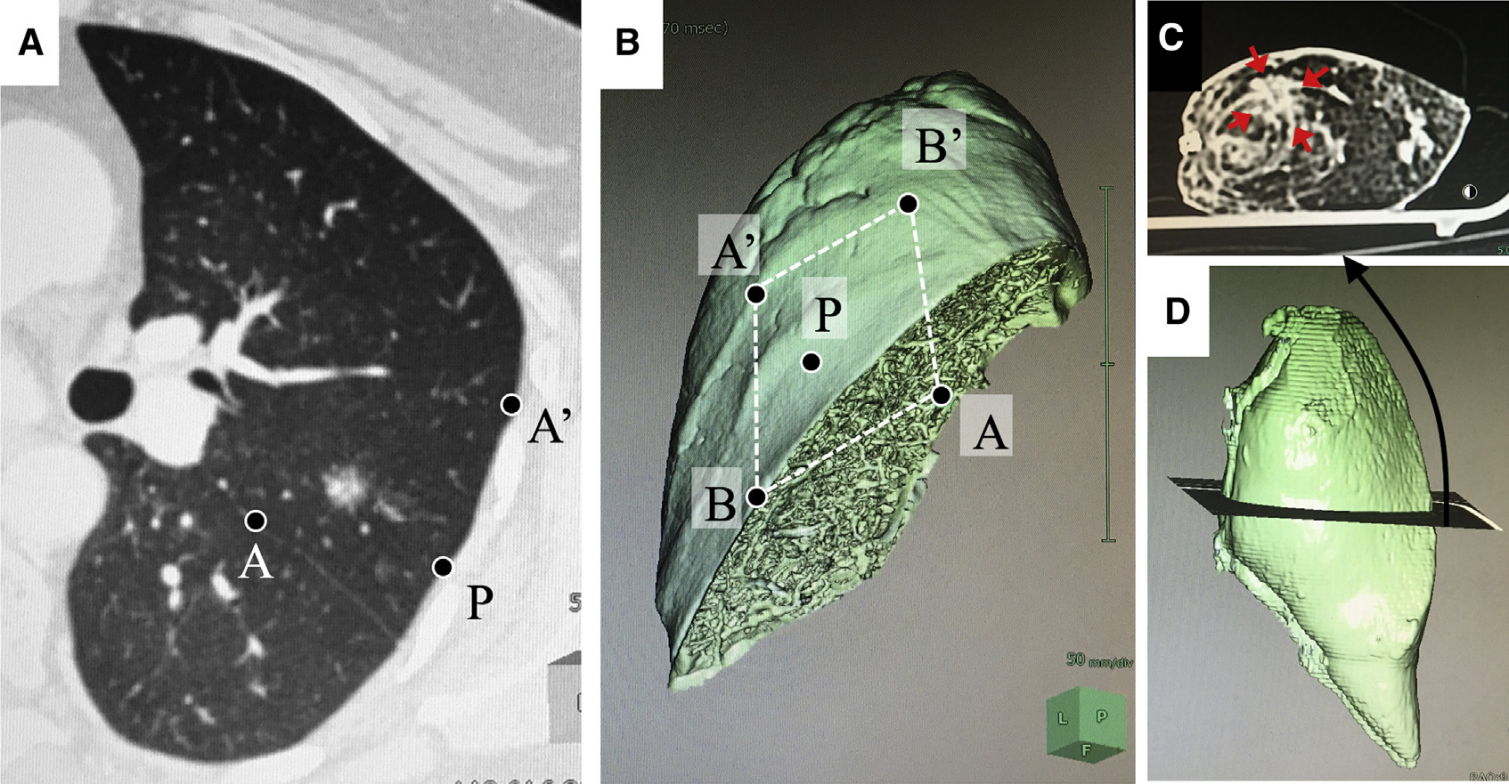
In patients with intrapulmonary subsolid lesion near the interlobar fissure (A), the tumor is resected together with the lung corner (B). In this situation, point A is located on the interlobar pleura (B). Although the lung lesion is not clearly palpable, computed tomography imaging of the resected specimen successfully depicted a subsolid lesion, yielding an accurate measurement of the surgical margin length (C). The intrapulmonary lesion is located at the center of the resected specimen (D).

In patients with intrapulmonary lesions near the pulmonary fissure or near the lung corner, the tumor was resected using the lung corner (Figure 5). In such situations, point A is located on the interlobar pleura (Figure 5). We resected the tumor together with the lung corner in 11 patients and did not resect the corner in 5 patients (Table 2). The locations of points A and A' were approximated in reference to some anatomical landmarks (apex, pulmonary vessels, interlobar fissure, etc). We generally used a flexible ruler for measurement of distances from the anatomical landmarks. According to the analysis of CBCT images for comparison of clipped sites with correct sites, the mean gap between the clipped points A (A') and correct points A (A') was 8 mm (range, 0-34 mm; Table 2), which were measured on a 3-dimensional volume-rendering lung model (Figure 3). We corrected the point A and A' on the basis of the CBCT, and the point P, B, and B' were subsequently marked on the basis of the corrected points A and A', yielding imaging of the cut lines in all patients.

**Table 2:** Intraoperative measurements

Case	CBCT acquisitions, times	Specimen CT	Resection type∗	Gap for A, mm	Gap for A', mm	Margin length after shrinkage, mm	Estimated margin length, mm
1	2	Yes	1	15	15	10	12
2	2	Yes	2	5	6	12	14.4
3	2	No	1	18	10	16	19.2
4	3	Yes	2	0	0	15	18
5	2	No	1	15	15	10	12
6	3	No	2	12	11	7	8.4
7	3	Yes	2	16	7	12	14.4
8	3	Yes	2	0	0	11	13.2
9	3	Yes	2	0	0	7	8.4
10	3	Yes	2	34	23	10	12
11	3	Yes	2	0	0	12	14.4
12	3	No	1	5	3	9	10.8
13	3	No	2	13	6	8	9.6
14	3	No	1	0	0	8	9.6
15	3	No	2	10	0	9	10.8
16	3	No	2	12	8	16	19.2

*CBCT*, Cone-beam computed tomography; *CT*, computed tomography; *Gap for A*, gap between the clipped point A and the correct point A; *Gap for A'*, gap between the clipped point A' and the correct point A'.

∗ Resection type 1 is wedge resection without lung edge; resection type 2 is wedge resection with lung edge.

**Figure E1:**
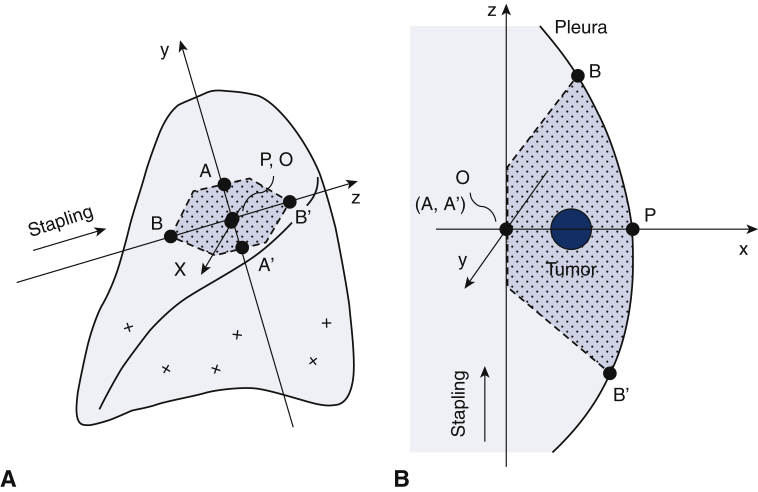
(A and B) Image of a hexagonal cut line for larger pulmonary resection using 3 or more cartridges. *AA*', the deepest point of the wedge resection; *BB*', direction of the stapling.

We performed wedge resection along the imaged cut lines in all the 16 patients. The target lesion was palpable in 8 patients and was not clearly palpable in the remaining 8 patients. The 8 impalpable specimens were scanned using conventional CT for confirmation of successful resection after instillation of air into the resected specimen. Thus, no patients required conversion to anatomical lung resection or conversion to open thoracotomy. According to the measured margin length, the mean margin length after shrinkage was 11 mm (range, 7-16 mm); thus, the estimated mean margin length (before shrinkage) was 13 mm (range, 8.4-19 mm). With the exception of 1 case, the estimated margin length was longer than the required margin length (maximum tumor diameter), suggesting that the surgical margin was satisfactory (Figure E1). In the 1 exception, the estimated margin length (9.6 mm) was slightly shorter than the maximum tumor diameter (11 mm; case 14 in Table 2; Figure E1). However, irrigation cytology to evaluate surgical stump positivity was negative in all 16 patients.

## Discussion

Although most previous studies focused only on the localization of intrapulmonary lesions using various modalities, in the current study, we focused on the use of CBCT in determining how to cut the lung to secure the required surgical margin. We proposed a new concept of imaging a cut line on the real lung on the basis of an imaging analysis of CBCT, which might contribute to reducing the stress on surgeons and patients. According to our preliminary experience, although we mainly investigated difficult cases (13 [81%] of the 16 patients had deep subpleural lesions, at a depth of ≥5 mm),^11^ we successfully resected all lesions with an acceptable surgical margin length without any open conversion. Although further experience is needed to verify our strategy, our method could be a valuable option for patients who undergo sublobar resection for potentially indistinct intrapulmonary lesions in institutions in which a hybrid operating room is available.

Sato and colleagues^6^ attempted to secure the surgical margin length by making multiple marks on the lung surface using blue dye during preoperative bronchoscopy, which might be advantageous for understanding the geometric position of subpleural lesions. Likewise, Finley and colleagues^2^ attempted to avoid missing target lesions using preoperative CT-guided marking with microcoils at the deep side of the lesion. The lesions were subsequently resected using combined intraoperative fluoroscopic and thoracoscopic visualization. Both procedures achieved satisfactory early outcomes. In contrast to these modalities, our method does not require a preoperative procedure, excessive radiation exposure (as described in the next paragraph), or the use of dedicated instruments.

With regard to radiation exposure, we generally obtained CBCT images a maximum of 2 or 3 times, including test scanning before the operation and confirmation scanning after resection, which are optional in clinical practice. Thus, we only obtained CBCT images once per patient for the purpose of adjusting the position of the pleural mark: we never repeated CBCT scanning to confirm whether the corrected markings were correct. Therefore, the amount of radiation exposure of our intraoperative procedure was within the acceptable limit.

We obtained a CT image of the resected specimen. According to our initial experience, we could clearly identify small intrapulmonary lesions within the resected specimen under the condition that the specimen was intentionally inflated with air.^13^ This imaging modality was particularly useful for patients with impalpable ground glass lesions, because without CT images we cannot intraoperatively judge the success of wedge resection. Although cutting the specimens into pieces can identify the presence or absence of the lesion within the specimen, it compromises the accurate measurement of the surgical margin, as well as the accurate evaluation of pathological invasiveness of the tumor because of tissue shrinkage. In our previous study, we clarified that the surgical margin length measured on CT was closely correlated with the length that was subsequently measured on formalin-fixed specimens (*r* = 0.984; *P* < .01; n = 12), although the length measured on CT imaging was significantly longer (20%) than the length measured on the formalin-fixed specimen.^13^ An additional benefit of this method might arise if the surgical margin is unfortunately insufficient and additional resection is needed: the CT image of the resected specimen might contribute to planning how to perform additional resection of the remaining lung lobe. In the current study, we used intraoperative CT of the resected specimen for 8 patients whose tumors were not clearly identified using direct palpation of the specimens (Table 2).

**Figure E2:**
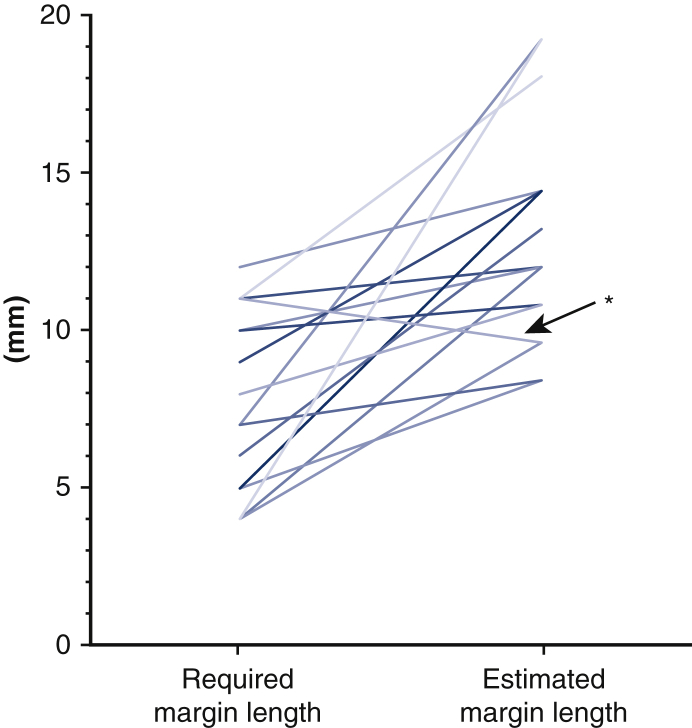
Comparison of the required margin length and the estimated margin length. With the exception of 1 case, the estimated margin length was longer than the required margin length (maximum tumor diameter) in all cases. In the one exception (*∗*), the estimated margin length (9.6 mm) was slightly shorter than the required margin length (11 mm).

We introduced a rhomboidal cut line in our representative patient who underwent wedge resection using 2 stapler cartridges. However, we can also image a hexagonal cut line if 3 or more cartridges are needed for deeper intrapulmonary lesions (Figure E2). In addition, regarding the direction of the stapler cut line (z-axis), the z-axis can be set in any direction during preoperative CT-based simulation. The setting of the z-axis is quite valuable because if the z-axis is in the wrong direction, it will compromise the handling of the autostapler, and furthermore, if the direction is incorrect, it will cause serious mass stapling of the lung tissue at the deepest wedge resection point (arc AOA' is simultaneously stapled). We recommend determining the direction of the z-axis so that the arc AOA' becomes shorter, which might be associated with less deformity of the remaining lung. Thus, for instance, if wedge resection is performed along the edge of the interlobar fissure, the z-axis should be set parallel to the interlobar fissure.

Because this report reflects our initial experience, the practical time required for the current procedure cannot be determined. However, we believe that experienced thoracic surgeons can perform preoperative CT-based simulation within 5-10 minutes during the induction of general anesthesia, and imaging of the cut line on the lung can be performed within 30 minutes, including the time required for trial and error adjustment using CBCT. Further experience is needed to verify our procedure.

An additional potential benefit of our preoperative simulation of wedge resection might be that we can estimate the thickness of the lung tissues to be stapled. During lung stapling, the arc AOA' is simultaneously stapled by the stapler; this is the thickest portion to be stapled. Using an image analysis software program, the thickness of the tissue can be estimated by measurement of the length of the arc AOA' and the mean CT density of the tissues along the arc AOA'. The estimation of the tissue thickness might be helpful for selecting the appropriate type of staple cartridge or for estimating the applicability of stapling in patients with pulmonary fibrosis.

The present study is associated with some limitations. First, the study population was relatively small. Therefore, the current patients did not have significant underlying lung diseases (eg, pulmonary emphysema or interstitial lung disease). The applicability of the current method on such patients should be clarified in the next study. Second, the simulation of wedge resection on preoperative CT and the determination of the intraoperative lung clipping sites were entirely directed by 1 experienced surgeon (K.U.). Thus, a multi-institutional study with a larger study population is needed to verify the reproducibility of the current study outcome.

## Conclusions

We established a novel procedure for imaging the cut line on the lung surface with intraoperative CBCT, which facilitated the performance of wedge resection with an acceptable surgical margin in patients with potentially impalpable intrapulmonary small lesions. Our method might be beneficial for patients and surgeons because it can be performed without preoperative intervention.

### Conflict of Interest Statement

The authors reported no conflicts of interest.

The *Journal* policy requires editors and reviewers to disclose conflicts of interest and to decline handling or reviewing manuscripts for which they may have a conflict of interest. The editors and reviewers of this article have no conflicts of interest.

## Supplementary Data

Video 1 A summary of thoracoscopic wedge resection of the left upper lobe along a cut line, determined using cone-beam computed tomography. Video available at: https://www.jtcvs.org/article/S2666-2507(22)00123-7/fulltext.

STROBE checklist
