# Supplementary figures and images for: Intraoperative cone-beam computed tomography to secure the surgical margin in pulmonary wedge resection for indistinct intrapulmonary lesions
Source: JTCVS Tech. 2022 Feb 23;13:219–28. doi: 10.1016/j.xjtc.2022.01.028 (PMC9196256; doi:10.1016/j.xjtc.2022.01.028)

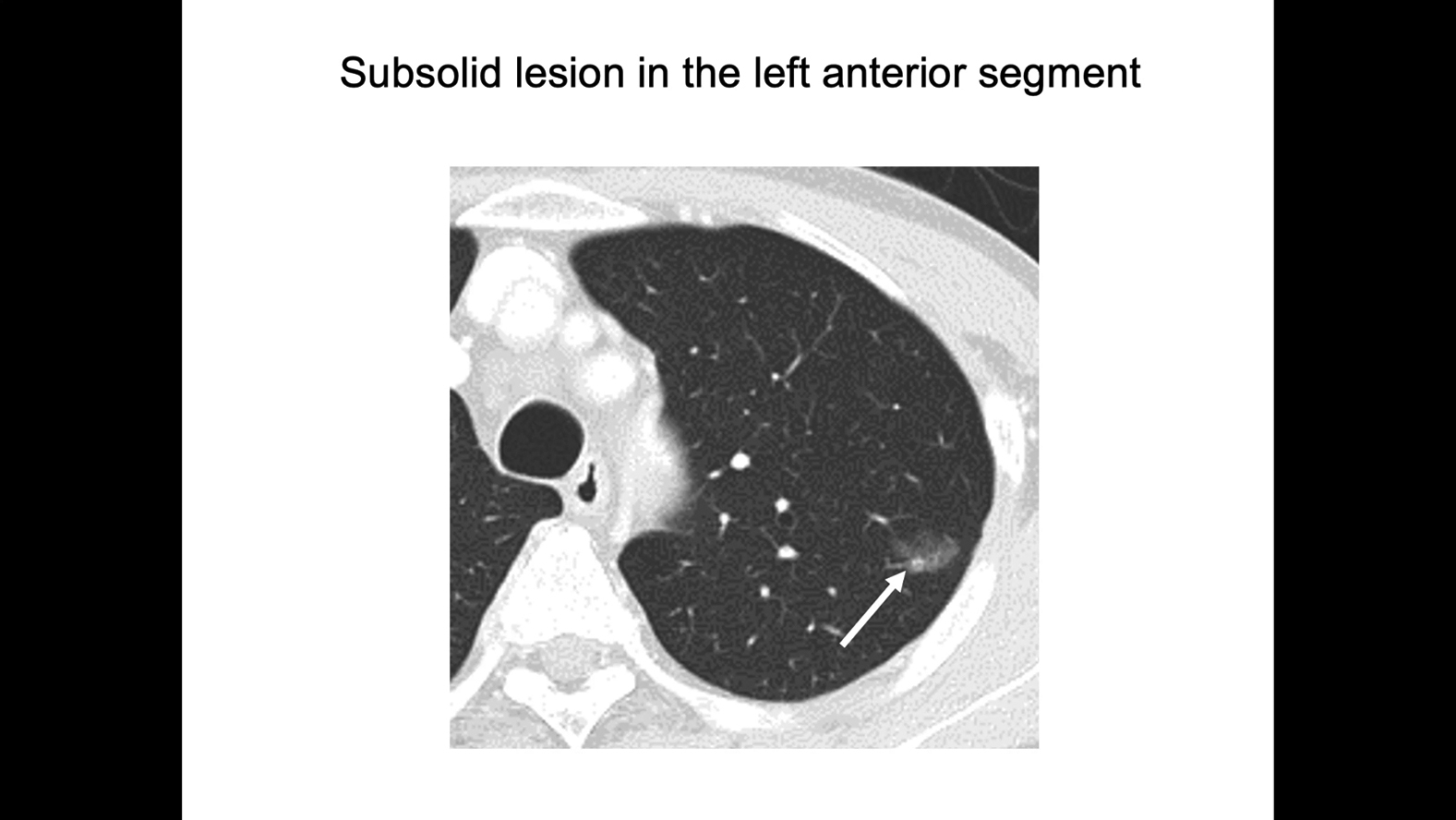

Supplement: Video 1 — A summary of thoracoscopic wedge resection of the left upper lobe along a cut line, determined using cone-beam computed tomography. Video available at: https://www.jtcvs.org/article/S2666-2507(22)00123-7/fulltext. [file fx2.jpg]
